# How enlightened self-interest guided global vaccine sharing benefits all: A modeling study

**DOI:** 10.7189/jogh.13.06038

**Published:** 2023-12-20

**Authors:** Zhenyu Han, Lin Chen, Qianyue Hao, Qiwei He, Katherine Budeski, Depeng Jin, Fengli Xu, Kun Tang, Yong Li

**Affiliations:** 1 Beijing National Research Center for Information Science and Technology (BNRist), Beijing, P. R. China; 2 Department of Electronic Engineering, Tsinghua University, Beijing, P. R. China; 3 Vanke School of Public Health, Tsinghua University, Beijing, P. R. China; 4 Institute for Healthy China, Tsinghua University, Beijing, P. R. China; 5 Nuffield Department of Medicine, University of Oxford, UK

## Abstract

**Background:**

Despite consensus that vaccines play an important role in combatting the global spread of infectious diseases, vaccine inequity is still a prevalent issue due to a deep-seated mentality of self-priority. We aimed to evaluate the existence and possible outcomes of a more equitable global vaccine distribution and explore a concrete incentive mechanism that promotes vaccine equity.

**Methods:**

We designed a metapopulation epidemiological model that simultaneously considers global vaccine distribution and human mobility, which we then calibrated by the number of infections and real-world vaccination records during the coronavirus disease 2019 (COVID-19) pandemic from March 2020 to July 2021. We explored the possibility of the enlightened self-interest incentive mechanism, which comprises improving one’s own epidemic outcomes by sharing vaccines with other countries, by evaluating the number of infections and deaths under various vaccine sharing strategies using the proposed model. To understand how these strategies affect the national interests, we distinguished imported from local cases for further cost-benefit analyses that rationalise the enlightened self-interest incentive mechanism behind vaccine sharing.

**Results:**

The proposed model accurately reproduces the real-world cumulative infections for both global and regional epidemics (R^2^>0.990), which can support the following evaluations of different vaccine sharing strategies: High-income countries can reduce 16.7 (95% confidence interval (CI) = 8.4-24.9, *P* < 0.001) million infection cases and 82.0 (95% CI = 76.6-87.4, *P* < 0.001) thousand deaths on average by more actively sharing vaccines in an enlightened self-interest manner, where the reduced internationally imported cases outweigh the threat from increased local infections. Such vaccine sharing strategies can also reduce 4.3 (95% CI = 1.2-7.5, *P* < 0.01) million infections and 7.0 (95% CI = 5.7-8.3, *P* < 0.001) thousand deaths in middle- and low-income countries, effectively benefiting the whole global population. Lastly, the more equitable vaccine distribution could help largely reduce the global mobility reduction needed for pandemic control.

**Conclusions:**

The incentive mechanism of enlightened self-interest we explored here could motivate vaccine equity by realigning the national interest to more equitable vaccine distributions. The positive results could promote multilateral collaborations in global vaccine redistribution and reconcile conflicted national interests, which could in turn benefit the global population.

Vaccines are a key resource for curbing the global spread of infectious diseases [1] through effective epidemic control at a relatively low economic cost compared with lockdowns or quarantine measures. The scarcity of vaccine resources during the early stage of disease outbreaks makes them a highly-demanded resource hoarded by high-income countries under the influence of self-priority policies. During the coronavirus disease 2019 (COVID-19) pandemic, more than three-fourths of the 7.8 billion manufactured vaccine doses were possessed by a few high-income countries as of 27 November 2021 [2], presenting “not a supply problem, but an allocation problem”, as stated by the World Health Organization (WHO). This inequal distribution of vaccines creates an environment for disease transmission in low- and middle-income countries and undermines epidemic control in high-income countries in this highly intertwined and globalised world [3-5].

International organisations have been trying to tackle this issue recently. The COVID-19 Vaccines Global Access (COVAX) Facility proposed a global risk-sharing mechanism for pooled procurement and equitable redistribution of vaccines in April 2020, through which it would purchase vaccines in bulk at a lower price and distribute them equitably among countries [6,7]. Although it aimed to provide at least two billion vaccine doses to cover 20% of each country's population in 2021 [7], it had only delivered less than half of the target doses by early 2022 [8,9]. One crucial reason for its failure is the absence of clear incentives for high-income participants to promote global vaccine sharing with their own wealth. According to the rational choice theory, the decisions made by individuals will maximise their own utility considering the corresponding costs and benefits [10]. Although it is ethically recognized that more equitable vaccine distribution is in “everyone's best interest” [11,12], a practical incentive with quantitative, real-world data supported evaluation is an important, yet missing piece for promoting global vaccine equity [13-15].

Motivated by the importance vaccine equity, scientists and public health researchers are striving to optimise the distribution of vaccine doses and understand its consequences. Recent studies focused on improving the effectiveness [16-18] and equity [19-23] of the domestic distribution of vaccines, but have been incapable of modeling the national interests of sovereign countries and scaling up to global vaccine distribution [11,24]. To address this gap, several early attempts employed highly simplified models with hypothetical parameters; however, they usually overlooked the structure of global mobility [20] and lacked evidence supported by real-world data [14,15,25,26]. Although they provide important theoretical results for various hypothetical scenarios, such studies cannot adequately reflect the complexity of real-world pandemics, limiting their capability of promoting global vaccine sharing with practical incentives.

We aimed to investigate the incentive mechanism of enlightened self-interest for global vaccine sharing through quantitative modeling analyses, where each party's willingness to share a portion of vaccines to assist others in return promotes their own interest. We do so by proposing a novel epidemiological model that provides a connected perspective of the world by simultaneously considering global vaccine distribution and human mobility. Through it, we evaluated a wide range of vaccine sharing strategies to explore the feasibility of the “enlightened self-interest” phenomenon, which could substantially motivate global cooperation in combating infectious diseases with equitable vaccine access. We assumed the shared vaccines are pooled globally and redistributed to all regions in proportion to their population sizes as COVAX, based on which enlightened self-interest could be achieved by balancing the two competing influence pathways of vaccine sharing and human mobility. We intended to propose and rationalise the practical incentive mechanism of enlightened self-interest that facilitates more equitable global vaccine distribution through a quantitative, real-world data supported modeling approach.

## METHODS

We conducted a modeling study that extended the classic epidemiological model (e.g. the susceptible, infectious, removed (SIR) model) [27] by considering regional mobility flows and vaccination processes. Leveraging the calibrated model on real-world infection records, we explored several vaccine sharing scenarios and their outcomes in terms of averted infections and deaths, aligned national interests with more equitable vaccine distribution. We use a one-way analysis of variance (ANOVA) to validate the statistical significance among the contrasting scenarios.

### Regional division and scenario setup

**Figure 1:**
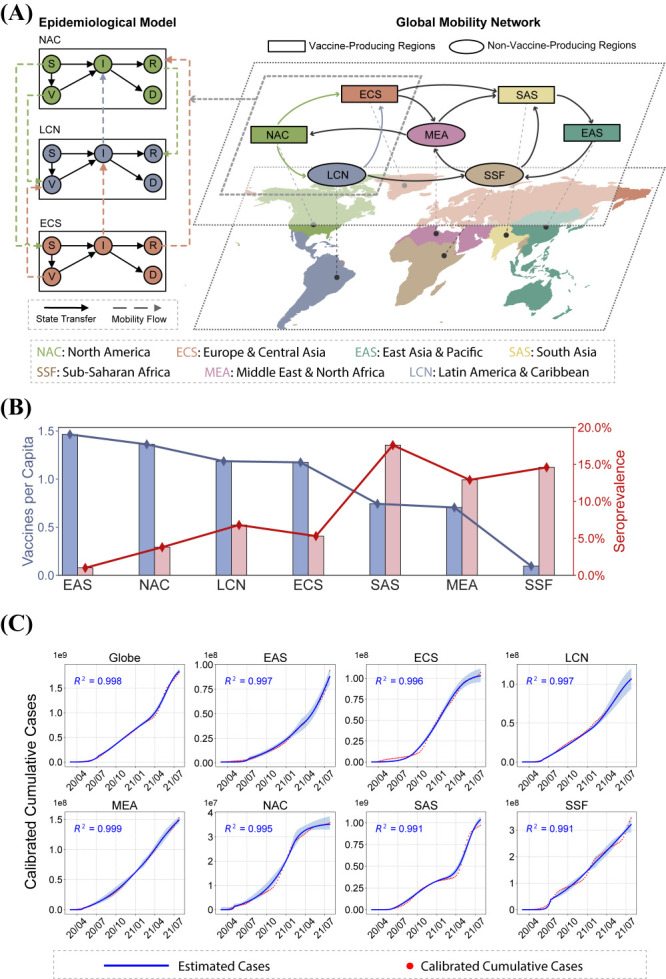
Model description and its performance in capturing real-world epidemic dynamics. **Panel A.** Illustration of our model framework. The globe is divided into 7 geographical regions, where each region consists of an individual epidemiological model considering breakthrough infections after vaccinated. People in different regions can move from one region to another, forming a global mobility network that characterized by the air traffic data from OAG. **Panel B.** Vaccines per capita and seroprevalence of COVID-19 in each region until 14 November 2021. Vaccine availability disobeys the epidemic situation in different regions. Specifically, SAS has the highest seroprevalence, while its vaccines per capita are much less than high-income regions such as NAC. **Panel C.** Result of model fitting in global and regional level, where shaded area represents the 99% confidence interval. We have R^2^>0.990 for all the regions, demonstrating the accuracy of our proposed model.

We used the COVID-19 pandemic to evaluate the existence and possible outcomes of a more equitable global vaccine distribution based on rich real-world data. Considering the varying epidemic situation and abilities to access vaccine supplies across the world, we divided the globe into seven regions according to the World Bank definitions [28]: East Asia and Pacific (EAS), Europe and Central Asia (ECS), Latin America and Caribbean (LCN), Middle East and North Africa (MEA), North America (NAC), South Asia (SAS), and sub-Saharan Africa (SSF) (**Figure 1**, Panel A and Table S1-S2 in the **Online Supplementary Document**). We categorised these regions into two groups based on their production capability (Text S1 in the **Online Supplementary Document**) [29,30], labeling EAS, ECS, NAC and SAS as “vaccine-producing regions” and LCN, MEA, and SSF (which lack manufacturing capability) as “non-vaccine-producing regions”. Since high-income countries have enough resources to secure their vaccine production capability, we assumed that the vaccine-producing regions mainly comprise high-income countries, while lower non-vaccine-producing regions mostly consist of lower-middle-income ones (Table S3 in the **Online Supplementary Document**).

### Global mobility-aware epidemiological model design

To capture the epidemic dynamics under different vaccine sharing strategies, we proposed a novel epidemiological model that systematically accounts for the breakthrough infection during vaccination process and the global mobility network. Specifically, each region maintains its own compartmentalised states for susceptible (*S*), vaccinated (*V*), infectious (*I*), recovered (*R*), and deceased (*D*) people. To reproduce real-world COVID-19 transmission with these compartmentalised states and avoid the strong assumption of permanent vaccination [15], we considered infections as a probabilistic process, where the vaccinated population has a reduced infection probability according to the current research [31-33], i.e. the breakthrough infection. We argue that breakthrough infection is necessary to accurately model the epidemic outcomes under different vaccine sharing strategies without overestimating their effectiveness, where both susceptible people and part of vaccinated people (considering the non-100% vaccine effectiveness) can get infected, which generates *I_s_* and *I_v_*, accordingly. In our model, the vaccinated infection cases tend to have a lower death rate [31-33], which affects the transfer possibility from infectious to recovered and deceased. The above contagion process in each region is characterized by the following ordinary differential equations:

d*S*_n_/d*t* = -*vcc*(*n*,*t*) - β*_n_*S*_n_*(I*_s_*_,_*_n_* + I*_v_*_,_*_n_*),

d*V_n_*/d*t* = *vcc*(*n*,*t*) - β*_n_*(1 - κ)*V_n_*(I*_s_*_,_*_n_* + I*_v_*_,_*_n_*),

d*I_s_*_,_*_n_*/d*t* = β*_n_*S*_n_*(I*_s_*_,_*_n_* + I*_v_*_,_*_n_*) - γ*_n_*I*_s_*_,_*_n_* - ψI*_s_*_,_*_n_*,

d*I_v_*_,_*_n_*/d*t =* β*_n_*(1 - κ)*V_n_*(I*_s_*_,_*_n_* + I*_v_*_,_*_n_*) - γ*_n_*I*_v,n_* - ψ(1 - σ)I*_v_*_,_*_n_*,

d*R_s_*_,_*_n_*/d*t* = γ*_n_*I*_s,n_*,

d*R_v_*_,_*_n_*/d*t* = γ*_n_*I*_v,n_*,

d*D_s_*_,_*_n_*/d*t* = ψI*_s_*_,_*_n_*

d*D_v_*_,_*_n_*/d*t* = ψ(1 - σ) I*_v_*_,_*_n_*

where S*_n_*, *V*_n_, *I_⋆,n_*, *R_⋆_*_,_*_n_*, *D_⋆_,_n_* are the susceptible, vaccinated, infected, recovered, and deceased people in region *n.vcc*(*n*,*t*) is the number of people who can be vaccinated in region *n* at time *t* derived by various vaccine sharing strategies. Specifically, we divide the infected, recovered and deceased people according to their vaccination status into *I_s,n_*, *R_s_*_,_*_n_*, *D_s_,_n_* and *I_v,n_*, *R_v_*_,_*_n_*, *D_v_,_n_* for unvaccinated and vaccinated people accordingly. It enables us to explicitly model the breakthrough infection process.

Based on these regional models, we overlayed a mobility network connecting these regions to form a global view of the pandemic. We used the international air traffic records from the Official Aviation Guide (OAG), which has proven to be highly informative for epidemic modeling [34], to reconstruct the global mobility network with all transportations. We could not acquire the detailed health status of passengers due to privacy concerns. The continued existence of asymptomatic COVID-19 cases makes it harder to distinguish the flow of infection cases [35], so we assumed infection cases move across regions proportionally to the prevalence of the source region. This assumption maximises the utility of real-world mobility data, while also allowing us to simulate the pandemic dynamics under different vaccine sharing strategies (Text S2 in the **Online Supplementary Document**).

### Characterizing different vaccine sharing strategies

We defined two parameters to characterize different vaccine sharing strategies: the “start-sharing point” and the “sharing percentage”. Each vaccine-producing region starts with only vaccinating its citizens until its vaccination rate reaches the “start-sharing point”. Then it starts to share the newly acquired vaccines by the “sharing percentage”. The shared vaccines are pooled globally and redistributed to all regions in proportion to their population sizes, which is consistent with the COVAX framework [36]. As such, we can design a series of typical vaccine sharing strategies by formulating different combinations of the “start-sharing point” (a) and the “sharing percentage” (b). For example, when b = 0%, vaccine-producing regions will not share any vaccine even after all their citizens are fully vaccinated, which we refer to as the “non-sharing” strategy. When a = 100%, b = 100%, the vaccine-producing regions will share all their newly produced vaccines after vaccinating their own citizens, denoted as the “selfish” strategy. Both the “non-sharing” and “selfish” strategies indicate the vaccine-producing region will prioritise their own citizens for full vaccination before they start sharing, which represents the typical self-priority behaviors. Conversely, an “altruistic” strategy means sharing 100% of their vaccine supplies from the beginning, where a = 0%, b = 100%. More fine-grained vaccine sharing strategies can be defined with specific combinations of a and b, ranging between the two extremes.

We evaluated the vaccine sharing strategies with different parameters and compared the corresponding number of infections or deaths in each region with the “selfish” strategy. We defined the feasible parameter combinations for “enlightened self-interest” guided vaccine sharing as those simultaneously which simultaneously reduce the infections in both vaccine-producing and non-vaccine-producing regions.

### Disentangling imported transmission and local transmission

New cases can be categorised as local transmission caused by the initial cases in the region and imported transmission caused by the inflow of infection cases and their descendant cases. To investigate how vaccine sharing strategies affect the epidemic outcomes in vaccine-producing regions and non-vaccine-producing regions, we need to disentangle these two types of transmission. Here we define imported transmission by summing up the number of imported infections and their subsequent infections and local transmission as infections caused by pre-existing local cases. We traced back the above process until December 2020, when COVID-19 vaccines were made available (Text S3 and Figure S5 in the **Online Supplementary Document**).

### Quantifying the impact of vaccine sharing on inter-regional mobility policy

We measured the mobility benefit of different vaccine sharing strategies as the equivalent mobility reduction under the “selfish” strategy by calculating the required reduction of inter-regional mobility under the “selfish” strategy. This was done to achieve a similar level of infection prevention as the inspected vaccine sharing strategies with no mobility constraint, which is considered as the equivalent mobility benefit of the given vaccine sharing strategy.

## RESULTS

### Reproducing epidemic developments under real-world vaccine distribution

We first showcased the significant global mismatch between COVID-19 prevalence, measured by seroprevalence statistics, and vaccine distribution in different regions (**Figure 1****,** Panels A and B). Regions with high seroprevalence possess few vaccine supplies, where nearly ten people share a single vaccine in SSF. By contrast, vaccine-producing regions such as EAS and NAC had the largest vaccine supplies, albeit with relatively lower seroprevalence. The mismatch between seroprevalence and vaccine distribution demonstrates the substantial vaccine inequity worldwide and corroborates the great potential for more equitable vaccine sharing.

To capture the above global mismatch and evaluate the epidemic outcomes under more equitable vaccine sharing strategies, we proposed a quantitative, real-world data supported modeling approach. Leveraging a metapopulation schema with compartmentalised states for susceptible (*S*), vaccinated (*V*), infectious (*I*), recovered (*R*), and deceased (*D*) people, we modeled the world into several regions interconnected by both vaccination sharing and population flows to capture both the spatial and temporal heterogeneity of the epidemiological severity and vaccine resources (Text S4 in the **Online Supplementary Document**). The proposed model accurately reproduces the vastly different and ever-changing epidemic dynamics in all the regions with R^2^>0.990 (**Figure 1****,** Panel C), which demonstrates sufficient capability to capture the disease transmission processes in a connected world.

### Enlightened self-interest guided vaccine sharing strategies

As an intuitive example, we attempted to reduce the mismatch between COVID-19 prevalence and vaccine distributions through “altruistic” strategy, where vaccine-producing regions would share all vaccine supplies to the global vaccine pool from the beginning. This naïve policy would lead to a reduction of 10.77% (95% CI = 10.38%-11.17%, *P* < 0.001) global infections compared to the “selfish” strategy (Figure S1 in the **Online Supplementary Document**), presenting a significant improvement in global pandemic outcomes, yet at the cost of incurring more infections in vaccine-producing regions, e.g. ECS suffers 4.37% (95% CI = 3.97%-4.74%, *P* < 0.001) more infections.

**Figure 2:**
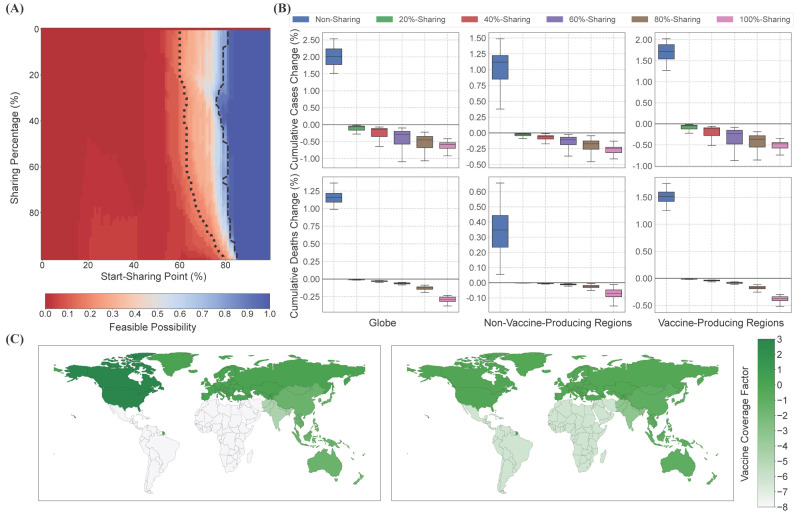
Impacts of enlightened self-interest guided vaccine sharing strategies. **Panel A.** Feasible parameter combinations of enlightened self-interest guided vaccine sharing strategies. The colors indicate the possibilities for each vaccine-producing region and the globe to have fewer infections compared with selfish strategy. Strategies that can achieve such simultaneous improvement over 80% possibility are noted as enlightened self-interest incentive mechanism. The boundary of 20% and 80% possibility are labeled by the dotted line and dashed line accordingly. **Panel B.** Increase of infections and deaths for different vaccine sharing strategies compared with selfish strategy around the globe, in the non-vaccine-producing regions and in the vaccine-producing regions. **Panel C.** Geographical vaccine distribution under the selfish strategy (left) and the 100%-sharing strategy (right). The vaccine coverage factor is calculated by the logarithm of the ratio between the number of vaccines and the population.

Such trade-offs can be avoided with more deliberated vaccine sharing strategies. The “start-sharing point” reaching 60% makes achieving “enlightened self-interest” possible (**Figure 2****,** Panel A). At a higher “start-sharing point”, countries with vaccine-sharing strategies are allowed to mare actively share vaccine supplies with other regions. As a conservative estimation, we also provided the parameter combinations that achieve “enlightened self-interest” over 80% likelihood (**Figure 2****,** Panel A). Under this strict definition, we still observed that, when the local vaccination rate reaches around 80%, vaccine-producing regions can still share most of their vaccine supplies with other regions. The “enlightened self-interest” guided vaccine-sharing strategy benefits both non-vaccine-producing and vaccine-producing regions (Figure S2 in the **Online Supplementary Document**).

We further evaluated how different levels of sharing, represented by the parameter “sharing percentage” (b), affect infections and deaths. Specifically, we sought the optimal “start-sharing point” for different “sharing percentages” to achieve their best pandemic outcomes in the feasible parameter combinations of “enlightened self-interest” guided strategies, noted as b%-Sharing accordingly. We further observed that a higher “sharing percentage” would result in a greater reduction in both infections and deaths for all kinds of regions (**Figure 2****,** Panel B). As an example, the “100%-sharing” strategy reduces 16.7 (95% CI = 8.4-24.9, *P* < 0.001) million infections and 82.0 (95% CI = 76.6-87.4, *P* < 0.001) thousand deaths in vaccine-producing regions, and 4.3 (95% CI = 1.2-7.5, *P* < 0.01) million infections and 7.0 (95% CI = 5.7-8.3, *P* < 0.001) thousand deaths in non-vaccine-producing regions. Moreover, 79.5% (95% CI = 76.9-87.5) of globally reduced infections occur to vaccine-producing regions, providing a strong incentive for proactively sharing vaccines. Simultaneously, the “non-sharing” strategy guided by extreme self-priority results leads to considerably more infections in every region, while EAS is impacted the most, with 14.8 (95% CI = 13.3-16.4, *P* < 0.001) million extra infections.

Furthermore, we compare the geographical distributions of vaccines under the “100%-sharing” strategy and the “selfish” strategy. Under the “selfish” strategy (**Figure 2****,** Panel C), non-vaccine-producing regions such as LCN and SSF possess few vaccine supplies. By contrast, under the “100%-sharing” strategy (**Figure 2****,** Panel C), non-vaccine producing regions receive vaccines without bringing great vaccine loss in vaccine-producing regions, which reduces the significant inequity in vaccine distribution. From the above, we demonstrated the feasibility of vaccine sharing strategies guided by the “enlightened self-interest” incentive mechanism, which could effectively relieve the pandemic outcomes for both vaccine-producing and non-vaccine-producing regions.

### Rationalising the “enlightened self-interest” incentive mechanism

**Figure 3:**
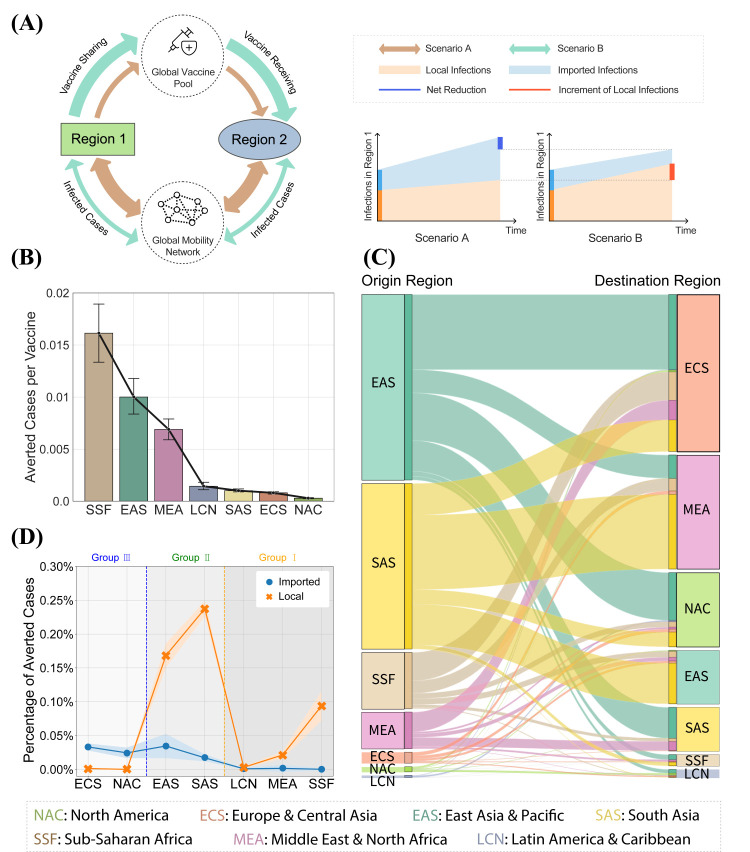
Rationalising the enlightened self-interest incentive mechanism. **Panel A.** Illustration of the incentive mechanism behind the enlightened self-interest guided vaccine sharing strategies. By categorising the infection into local infections (orange area) and imported infections (blue area), we expect under more active vaccine sharing strategies, there will be a net reduction in infections (deep blue line), although the local infections may increase (red line), due to the reduced imported infections. **Panel B.** Marginal utility of vaccines based on real-world vaccine distribution. It demonstrates that the effectiveness of extra vaccines vanishes in regions with high vaccination rates, such as NAC and ECS. The error bar demonstrates 95% of confidence intervals. **Panel C.** Flow of reduced infection cases under the enlightened self-interest guided vaccine sharing strategy, started from 90% vaccination with 90% vaccines shared, comparing with the selfish strategy. **Panel D.** Quantifying the disentangled imported transmission and local transmission. For group 1, they have reduced local transmission due to vaccine sharing. For group 2, both the imported transmission and local transmission will reduce. For group 2, the received imported transmission benefit outweighs the increasing of local transmission. The shaded area represents the standard deviation.

Having shown the feasibility of vaccine sharing strategies that benefit all regions, we further explored how such mutual benefits are achieved. The epidemic dynamics of different regions are coupled via two competing influence pathways: vaccine sharing and inter-regional mobility (**Figure 3****,** Panel A). As for vaccine sharing, vaccine-producing regions would share their vaccines with non-vaccine-producing regions, which could improve the global vaccine equity but might also increase their own local epidemic risk. However, due to the second pathway of highly intertwined inter-regional mobility, vaccine sharing might in return also benefit the vaccine-producing regions.

To validate the above hypothesis, first we evaluated the marginal utility of vaccines, i.e. the averted cases per extra vaccine. We found that the marginal utility of vaccines is significantly higher in non-vaccine-producing regions due to the severe shortage, where, for example, the averted cases per vaccine in SSF is 79.2 (95% CI = 58.2-100.1, *P* < 0.001) times of NAC (**Figure 3**, Panel B). In fact, we observe a strong negative correlation (Spearman R = -0.429) between the vaccination rate and the averted cases per extra vaccine (Supplementary Method in the **Online Supplementary Document**). Therefore, due to the inter-regional mobility, the higher marginal utility of vaccines in non-vaccine-producing regions indicates that vaccine sharing could reduce imported cases in return for vaccine-producing regions by maximising the potential value of limited vaccine supplies, achieving *“*enlightened self-interest”.

We evaluate how the second influence pathway, i.e. the inter-regional mobility, affects vaccine-producing regions. Specifically, we simulate the flow of reduced infection cases under the “enlightened self-interest” guided sharing strategy compared with the “selfish” strategy in **Figure 3****,** Panel C. We observe a significant reduction of imported cases in ECS and NAC, which accounts for 49.7% (95% CI = 48.8%-50.6%, *P* < 0.001) in all regions. Such benefits for vaccine-producing regions are mainly achieved from reduced infection cases in EAS, SAS, SSF and MEA. Except for EAS, where its large population base contributes to the reduction of infected inter-regional travelers, these regions have relatively fewer vaccines per capita (**Figure 3****,** Panel B). The significantly reduced imported cases in vaccine-producing regions compensate for or even benefit their vaccine sharing behaviors (Figures S3-S4 in the **Online Supplementary Document**). These results suggest the disproportionate COVID-19 vaccine effect and strong inter-regional mobility coupling combined to facilitate the vaccine sharing strategies that meet the vaccine-producing regions' “enlightened self-interests”, where the vaccine-producing regions are in fact better off by sharing vaccines globally instead of adopting the “selfish” strategy.

To further quantify the trade-off between the competing influence pathways of vaccine sharing and inter-regional mobility, we disentangled the averted local transmission and imported transmission with respect to the “selfish” strategy as the measurements for our proposed “enlightened self-interest” guided vaccine sharing strategy (see Method in the **Online Supplementary Document** for details). Weobserved all regions had a net positive benefit in imported transmission since the global COVID-19 infection is reduced by vaccine sharing (**Figure 3**, Panel D). Moreover, the vaccine-producing regions (ECS, NAC, EAS, and SAS) received the most benefit from reduced imported transmission as the main destination of global mobility flow (Figure S3 in the **Online Supplementary Document**). The non-vaccine-producing regions in group 1 (LCN, MEA, and SSA) benefited more from local transmission than imported transmission under the “enlightened self-interest” guided vaccine sharing strategy, because they gain access to more globally pooled vaccines. Group 2 were regions where both the imported and local transmission are reduced substantially and which comprised large population and relatively small vaccine production capabilities (EAS and SAS). They would therefore actually receive more vaccines from the global pool albeit being vaccine-producing regions. As a result, the averted case in local transmission is 6.65 (95% CI = 5.28-9.02, *P* < 0.001) and 15.1 (95% CI = 13.7-16.5, *P* < 0.001) times of the imported transmission accordingly. For group 3 of NAC and ECS, the change in local transmission is relatively small, because they are the main regions that give out extra vaccines with low margin utility. However, the benefit of reduced imported transmission outweighed the slight increase in local transmission by over 32.9 (95% CI = 27.5-38.2, *P* < 0.001) times, which aligned vaccine sharing with their “enlightened self-interest”. Therefore, we demonstrated a feasible incentive mechanism to promote equitable global vaccine redistribution driven by “enlightened self-interest”.

### Equitable vaccine sharing promotes a connected world

During the pandemic, most parts of the world have implemented various travel restriction policies, which significantly reduced the inter-regional mobility by approximately 80.7% (measured by the number of travelers). As another incentive for more equitable vaccine distribution, we examine how active vaccine sharing promotes global connectivity without worsening the epidemic outcomes.

**Figure 4:**
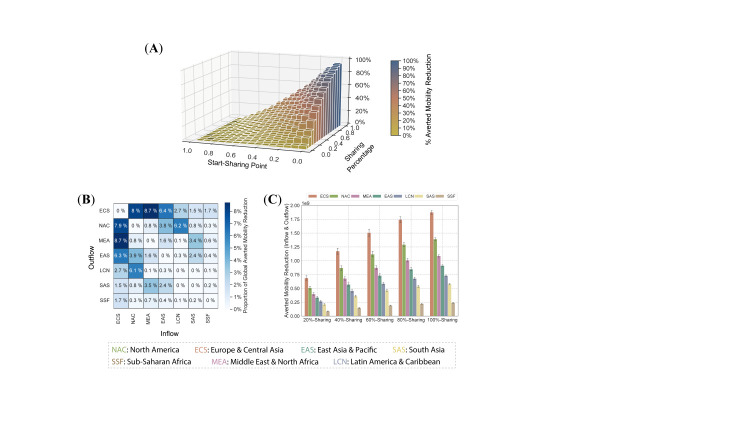
Active vaccine sharing promotes inter-region mobility. **Panel A.** Percentage of averted reduction in mobility under different vaccine sharing strategies. A more active sharing can prevent more mobility reduction, where up to 94.8% (95% CI = 94.3-95.3, *P* < 0.001) of mobility reduction can be averted. **Panel B.** Proportion of averted reduction in global mobility for each region, under the enlightened self-interest guided vaccine sharing strategy. **Panel C.** Volume of averted reduction in mobility under different enlightened self-interest guided vaccine sharing strategies, where error bars represent the 99% confidence interval. From Panel B and Panel C, we can find vaccine-producing regions, such as ECS and NAC, are expected to benefit most from mobility recovery in both relative proportion and absolute mobility volume.

Intuitively, a more equitable global vaccine distribution can reduce the infection risk embedded in the inter-regional population flow. A more “altruistic” sharing strategy in which vaccine sharing starts earlier and the sharing percentage is larger, can facilitate a higher level of inter-regional mobility, which can prevent up to 94.8% (95% CI = 94.3%-95.3%, *P* < 0.001) of mobility reduction (**Figure 4**, Panel A).

Moreover, most of the averted mobility reduction is associated with the vaccine-producing regions (**Figure 4****,** Panel B). NAC and ECS account for 48.8% (95% CI = 48.3%-49.3%, *P* < 0.001) of the total prevented mobility inflow reduction around the world, and EAS also accounts for 15.3% (95% CI = 15.2%-15.5%, *P* < 0.001) of prevented mobility inflow reduction due to its large population. We further validate this finding across different sharing strategies, where vaccine-producing regions constantly benefit more than non-vaccine-producing regions (**Figure 4****,** Panel C). Major vaccine-producing regions (NAC and ECS) benefited most under different “enlightened self-interest” guided vaccine sharing strategies in terms of averted mobility reduction. Specifically, more than 3.21 (95% CI = 3.16-3.27, *P* < 0.001) billion inter-regional travelers can move freely under “100%-sharing” strategies to NAC and ECS. These observations on global mobility provide yet another incentive for vaccine-producing regions to actively share vaccine supplies with the world, which suggests they can also benefit more in the transition back to a more connected world.

## DISCUSSION

We proposed a novel epidemiological model that can simultaneously model global vaccine distribution and real-world human mobility, which exemplifies how different vaccine sharing strategies affect the connected world. We quantitatively demonstrated the existence and potential value of “enlightened self-interest” incentive mechanism during COVID-19 pandemic, which can promote global vaccine sharing without harming the interest of vaccine-producing regions.

### Principal findings

Our epidemiological model goes beyond existing research that adopts a once-and-for-all vaccination assumption by explicitly considering the breakthrough transmission process for vaccinated people. Combined with the global mobility network reconstructed from the OAG international air traffic records, we successfully reproduce real-world COVID-19 epidemic dynamics with R^2^>0.990 on both global and regional scales. Our model provides a solid real-world basis for evaluating practical vaccine sharing strategies, which is an important advantage compared with existing research. As an example, Wagner et al. [14] constructed a highly simplified scenario with only two regions without fitting to real-world epidemic, which forbids them to propose a practical vaccine sharing strategy that suits the real-world scenario with complicated international mobility. Similarly, Lampert et al. [13] proposed a game theory approach to identify the condition for hypothetical vaccine-rich countries to share their extra vaccines, while the lack of epidemiological models weakens their credibility. Rotesi et al. [15] found that, after reaching herd immunity, the USA could donate vaccine doses to other countries without harming their own interest. However, the absence of real-world mobility patterns weakens the conclusions of these studies, and the absence of breakthrough infection processes makes it fails to provide concrete incentives for vaccine-rich countries. Going beyond these important theoretical works in hypothetical scenarios, our model enables the practical policy design rooted in strong real-world data.

Specifically, our model maps the vaccine distributions to their potential outcomes, supporting the quantitative evaluation of “enlightened self-interest” incentive mechanism. Through it, we showcased the feasibility of “enlightened self-interest” in COVID-19 vaccine sharing, where vaccine-producing regions can share their vaccines with other parts of the world after nearly 80% of their domestic people are vaccinated. Contradicting the common idea that, by sharing vaccines, a country must sacrifice its self-interest, we highlight that the vaccine sharing guided by the “enlightened self-interest” incentive mechanism can relieve epidemic outcomes in the vaccine-producing regions, which even surpass those in non-vaccine-producing regions. This phenomenon provides strong incentives for active vaccine sharing where the pandemic outcomes in both vaccine-producing regions and non-vaccine-producing regions can be simultaneously improved. We provided a larger feasible region for safely sharing vaccines with high credibility than existing works [13-15], where the global mobility enables quick action in promoting vaccine equity and the consideration of breakthrough infection guarantees conservative analyses. Furthermore, we demonstrated the mechanism behind such win-win vaccine sharing strategies, where the benefit from reduced internationally imported cases outweighs the threat from increased local infections due to the disproportionate vaccine effect and highly intertwined global mobility. Finally, we evaluate the benefit of active vaccine sharing in terms of global connectivity. While the strong travel restriction policies might be necessary to curb the pandemic, they also considerably disrupted global supply chains and undermined the global economy [37,38]. However, simply lifting these policies might lead to unexpected epidemic resurgence. By more actively sharing vaccine resources under “enlightened self-interest” incentive mechanism, up to 94.8% (95% CI = 94.3%-95.3%, *P* < 0.001) of inter-regional mobility reduction can be averted compared with the “selfish” strategy, promoting the free movements of talents and goods [39-41] in these regions. Under a more equitable global vaccine distribution, vaccine-producing regions could maintain a higher level of inter-regional mobility, providing another incentive for actively sharing vaccines.

Leveraging the proposed modeling approach, several different allocation problems can be solved to promote more equitable resource distribution driven by “enlightened self-interest”. Specifically, we summarized three different scenarios that can be solved by the proposed model with necessary adaptions. First, the medicines. Similar to vaccine resources for COVID-19, equitably distributing drugs for other infectious diseases is also critical to mitigating the possible global crisis, such as ZMapp for Ebola [42]. Considering the similarity in cost and efficacy of vaccines and medicines, our model can be extended to the medicine allocation problem with few modifications. Second, the medical equipment. Different from vaccines and medicines that usually have a high research and development cost but a low production cost, medical equipment has a high price and is not intended for one-time use. Besides, the benefit of medical equipment is not to reduce the possible transmission, but to resecure severe patients. We can adapt the epidemiological model to capture the imbalance between patient demand and the availability of medical equipment in terms of time and space, which further informs the years of potential life lost (YPLL) or human capital metrics [43] to solve the allocation problem from the “enlightened self-interest perspective”. Third, the health workforce. By organizing international medical teams and flexibly deploying the health workforce, the potential impact of epidemic outbreaks can be greatly mitigated [44]. Unlike the distribution of high-value medical equipment, the cost of scheduling health care personnel is much lower. However, due to the long training procedure required for skilled health care professionals, the cost of potential loss is extremely high. With proper modeling approaches to quantify the cost and benefit, we can deploy the proposed model in a series of allocation tasks.

### Limitations

Our study has several limitations. First, our proposed model adopts a classic compartmental framework, which might simplify real-world epidemic dynamics. The primary source of uncertainties lies in the estimation of epidemiological parameters, ie, the infection rate and recovery rate of COVID-19. We propose a 3-fold solution to capture these uncertainties. First, we adopt a metapopulation framework that fits different sets of parameters for each region, accounting for the spatial heterogeneity. We then divide the simulation period into three phases according to the mutation timeline (Table S6 in the **Online Supplementary Document**), accounting for the temporal heterogeneity. Finally, we capture the randomness of the parameters by 40 random initialisations of the model, which generates CIs in all analyses. However, it still relies on several classic assumptions. As an example, the countries within each region in the same phase share the same epidemiological parameters, and the vaccine effectiveness is constant across time. Besides, we also assume the epidemiological parameters are decoupled from different vaccine sharing strategies. More fine-grained behavior and demographic data are needed to challenge these conventional assumptions. Since our model is proven effective in tracing global epidemic development, we leave the more fine-grained epidemic model as a future work.

Second, we adopt a relatively straightforward strategy in redistributing the shared vaccine doses according to regional population, without considering the capacity of medical infrastructures. However, this redistributing strategy is in accordance with the COVAX project, which is easier to be implemented in the real world. Nevertheless, with such a redistribution scheme we are still able to reveal the feasibility of a more equitable vaccine distribution, which in turn emphasizes the robustness of our findings. Our study provides general evidence that we can achieve such Pareto improvement and a more equitable vaccine distribution, based on which more detailed, fine-grained vaccine distribution can be explored by introducing more advanced modeling assumptions. To further magnify the benefit, future studies are needed to devise more targeted redistribution schemes based on the epidemic dynamics and detailed profiles of each region.

Third, we focus on how to share vaccines globally, without probing into the domestic distribution of vaccines. For domestic distribution, several studies have provided important insight [17,19,45]. However, the global vaccine distribution received woefully inadequate research attention due to the difficulty of reconstructing the worldwide pandemic, even though this problem is becoming increasingly important. With the enlightenment of “enlightened self-interest”, we have proved both the possibility and the importance of equitable vaccine sharing at the global level, and it is worth putting future efforts to combine these two perspectives of vaccine sharing. With simultaneous consideration of both domestic and global vaccine distribution, we can form a hierarchical vaccine distribution framework, providing more fine-grained insights for vaccine sharing.

Fourth, we noticed the possible reliability issue of COVID-19 case statistics during the early outbreaks of COVID-19 [46,47]. To minimise these errors, we refer to the most comprehensive systematic review of COVID-19 seroprevalence, which contains 968 studies with more than 9.3 million participants in 74 countries [48]. It helps to preclude a large percentage of studies with insufficient reliability (496, 51% of all the studies) and to provide cross-validated, corrected median seroprevalence data. Nevertheless, there may still be certain flaws in the case estimation during the early stage of the pandemic.

## CONCLUSIONS

Here we proposed an accurate epidemiological model that simultaneously considers global vaccine distribution and human mobility, acting as a powerful tool for us to examine the feasibility and effectiveness of global vaccine sharing. Based on the proposed model, we highlighted the surprising fact that the “enlightened self-interests” of high-income countries are aligned with more equitable global vaccine distribution. We showed that, during a pandemic such as COVID-19, high-income countries should start sharing their extra vaccines after when nearly 80% of their citizens are vaccinated. To promote vaccine equity, countries should not criticize from a moral high ground, but to provide tangible incentives for action. Our findings enlighten such incentives to promote multilateral collaborations in global vaccine redistribution and vaccine equity, which facilitates a mutually benefiting world, supporting the thesis that, in combatting the pandemic, “no one is safe until everyone is safe” [49].

## Additional material


Online Supplementary Document
