# Supplementary material for: How enlightened self-interest guided global vaccine sharing benefits all: A modeling study
Source: J Glob Health. 2023 Dec 20;13:06038. doi: 10.7189/jogh.13.06038 (PMC10731134; doi:10.7189/jogh.13.06038)
Supplement: Online Supplementary Document [file jogh-13-06038-s001.pdf]

Supplementary information of How enlightened self-interest guided global vaccine sharing benefits all: a modeling study

Zhenyu Han, Lin Chen, Qianyue Hao, Qiwei He, Katherine Budeski, Depeng Jin, Fengli Xu, Kun Tang and Yong Li

Kun Tang and Yong Li are the corresponding author.
E-mail: tangkmail.tsinghua.edu.cn, liyong07tsinghua.edu.cn

Supplementary Datasets

In this work, we use six datasets for model fitting and vaccine distribution strategies modeling: COVID-19 confirmed cases data, COVID-19 vaccine administration estimates, COVID-19 vaccine production estimates, global flight records and population distribution data.

A) COVID-19 confirmed cases data: To depict the epidemic transmission in each region, we adopt the COVID-19 confirmed cases time-series data released by Johns Hopkins University[1]. The data is available at https://github.com/CSSEGISandData/COVID-19. We aggregate the country (entity) level cumulative infections into regions according to the taxonomy of World Bank[2].

B) COVID-19 seroprevalence data: The above case records contain the official case statistics from the governments, where non-negligible errors due to the limited testing capacity and wrong statistic models in the early stage of the outbreak have been identified[3,4]. To minimize these errors, we adopt seroprevalence studies to calibrate the case data. According to the largest review of COVID seroprevalence research[5] that contains 968 studies with more than 9.3 million participants, we preclude highly unreliable ones estimated and use the corrected median seroprevalence provided by the authors to reduce the potential bias.

C) COVID-19 vaccine administration estimates: We use COVID-19 vaccine doses administered estimates released by Our World in Data[6]. It contains the detailed number of cumulative vaccine does administered in each country (entity) by day. It provides a real-world basis for calibrating our model to capture the disease transmission patterns that support the exploration of more equitable vaccine sharing strategies. This data is widely acknowledged, and has been used in a series of high-quality studies[7,8]. It is available at https://ourworldindata.org/grapher/cumulative-covid-vaccinations.

D) COVID-19 vaccine production estimates: We use the COVID-19 vaccine producing capacity estimates released by Statista[9]. It contains the total number of COVID-19 vaccine doses produced by each country (entity) till 2021-03-03. We only care about the vaccine production shares to trace back the origins of the administrated vaccines. Therefore, the error in the specific number of production estimates is acceptable. It is available at https://www.statista.com/chart/24492/total-covid-19-vaccine-production-by-country/.

E) Global flight data: We purchased the global flight records from the Official Aviation Guide (OAG). It has the full volume seat order data collected from the International Air Transport Association (IATA), the official organization that regulates the global airline industry. This data has also been widely acknowledged and adopted in impactful studies by various researchers[10–14]. We obtain all the international flight orders in country (entity) level from January 2019 to July 2021. During data processing, we aggregate the flight orders into World Bank regions.

F) Population distribution data: We use World Bank statistics about global population in our model, which is available at https://data.worldbank.org/indicator/SP.POP.TOTL.

Supplementary Text

Text S1. Estimation of vaccine producing capacity by time

We estimate the daily COVID-19 vaccine produced capacity of each vaccine-producing region out of the COVID-19 vaccine doses administered data by Our World in Data[6] and the COVID-19 vaccine production data by Statista[9], which are shown in Fig. SS6. We assume that the total number of vaccines produced by the vaccine-producing regions daily is identical to those administered by all regions around the world in the COVID-19 vaccine doses administered data. The cumulative total number of produced (administered) vaccines is shown in Fig. SS6A. We obtain the vaccine production shares of various countries till

2021-03-03, which is shown in Fig. SS6B. We consider countries producing more than 10 million doses of vaccines to be vaccine-producing-able ones and the aggregated vaccine production shares into regions are shown in Fig. SS6C. We assume that the shares keep unvaried and the vaccine producing capacity by time can be estimated by multiplying the shares and the cumulative total number of produced vaccines.

Text S2. Epidemiological model design

As demonstrated in Methods, we propose a novel epidemiological model that considers the breakthrough infection and global mobility. For each region, we have the following equations:

$$\frac{\mathrm{d}S_n}{\mathrm{d}t} = -vcc(n,t) - \beta_n S_n(I_{s,n} + I_{v,n}), \tag{1}$$

$$\frac{\mathrm{d}V_n}{\mathrm{d}t} = vcc(n,t) - \beta_n(1-\kappa)V_n(I_{s,n} + I_{v,n}), \tag{2}$$

$$\frac{\mathrm{d}I_{s,n}}{\mathrm{d}t} = \beta_n S_n(I_{s,n} + I_{v,n}) - \gamma_n I_{s,n} - \psi I_{s,n}, \tag{3}$$

$$\frac{\mathrm{d}I_{v,n}}{\mathrm{d}t} = \beta_n(1-\kappa)V_n(I_{s,n} + I_{v,n}) - \gamma_n I_{v,n} - \psi(1-\sigma)I_{v,n}, \tag{4}$$

$$\frac{\mathrm{d}R_{s,n}}{\mathrm{d}t} = \gamma_n I_{s,n}, \tag{5}$$

$$\frac{\mathrm{d}R_{v,n}}{\mathrm{d}t} = \gamma_n I_{v,n}, \tag{6}$$

$$\frac{\mathrm{d}D_{s,n}}{\mathrm{d}t} = \psi I_{s,n}, \tag{7}$$

$$\frac{\mathrm{d}D_{v,n}}{\mathrm{d}t} = \psi(1-\sigma)I_{v,n}, \tag{8}$$

where $S_n, V_n, I_{\star,n}, R_{\star,n}, D_{\star,n}$ are the susceptible, vaccinated, infected, recovered and deceased people in region n. Specifically, we divide the infected, recovered and deceased people according to their vaccination status into $I_{s,n}, R_{s,n}, D_{s,n}$ and $I_{v,n}, R_{v,n}, D_{v,n}$ for unvaccinated and vaccinated people accordingly. It enables us to explicitly model the breakthrough infection process.

Equation (1) demonstrates the change in susceptible people due to the vaccination process (the first term on the right side) and infection process (the second term on the right side). $vcc(n,t)$ is the number of people who can be vaccinated in region n at time t derived by various vaccine sharing strategies. For the infection process, both $I_{s,n}$ and $I_{v,n}$ can lead to new infections with infection rate β_n. We follow the implicit assumption in the classic SIR model that people in the same region are homogeneously mixed, which generates the term $-\beta_n S_n(I_{s,n} + I_{v,n})$. The infected people from state S_n will transit to state $I_{s,n}$, as shown in Equation (3). $I_{s,n}$ will recover according to the recovery rate γ_n or die according to the death rate ψ_n, as the second and the third term in Equation (3) right side shows. The recovered people and dead people from $I_{s,n}$ turn to $R_{s,n}$ and $D_{s,n}$ accordingly in Equation (5) and (7). For vaccinated people, we have a similar process but with extra vaccine effectiveness parameters towards infection and death, which is denoted as κ and σ accordingly. The state transfer processes for vaccinated people are shown in Equation (4), (6) and (8). Details about model parameters can be found in Supplementary Table 4.

Based on the above setting, we formally introduce the global mobility network as the following equations:

$$\text{InputFlow}_{S_n}(t) = \sum_{m,m\neq n} mobility_{m,n}(t) \times \frac{S_m}{N_m}, \quad \text{OutputFlow}_{S_n}(t) = \sum_{m,m\neq n} mobility_{n,m}(t) \times \frac{S_n}{N_n}; \tag{9}$$

$$\text{InputFlow}_{V_n}(t) = \sum_{m,m\neq n} mobility_{m,n}(t) \times \frac{V_m}{N_m}, \quad \text{OutputFlow}_{V_n}(t) = \sum_{m,m\neq n} mobility_{n,m}(t) \times \frac{V_n}{N_n}; \tag{10}$$

$$\text{InputFlow}_{I_{s,n}}(t) = \sum_{m,m\neq n} mobility_{m,n}(t) \times \frac{I_{s,m}}{N_m}, \quad \text{OutputFlow}_{I_{s,n}}(t) = \sum_{m,m\neq n} mobility_{n,m}(t) \times \frac{I_{s,n}}{N_n}; \tag{11}$$

$$\text{InputFlow}_{I_{v,n}}(t) = \sum_{m,m\neq n} mobility_{m,n}(t) \times \frac{I_{v,m}}{N_m}, \quad \text{OutputFlow}_{I_{v,n}}(t) = \sum_{m,m\neq n} mobility_{n,m}(t) \times \frac{I_{v,n}}{N_n}; \tag{12}$$

$$\text{InputFlow}_{R_{s,n}}(t) = \sum_{m,m\neq n} mobility_{m,n}(t) \times \frac{R_{s,m}}{N_m}, \quad \text{OutputFlow}_{R_{s,n}}(t) = \sum_{m,m\neq n} mobility_{n,m}(t) \times \frac{R_{s,n}}{N_n}; \tag{13}$$

$$\text{InputFlow}_{R_{v,n}}(t) = \sum_{m,m\neq n} mobility_{m,n}(t) \times \frac{R_{v,m}}{N_m}, \quad \text{OutputFlow}_{R_{v,n}}(t) = \sum_{m,m\neq n} mobility_{n,m}(t) \times \frac{R_{v,n}}{N_n}; \tag{14}$$

where the $\text{InputFlow}_{\{\star_n\}(t)}$ and $\text{OutputFlow}_{\{\star_n\}(t)}$ represent the number of people in state $\star$ that move in/out from region n. The summation in equation is over each region. The $mobility_{m,n}(t)$ depicts the total population flow from region m to region n at time t, which is set according to the calibrated global mobility data. N_m depicts the total population in region m, which equals to $S_m + V_m + I_{s,m} + I_{v,m} + R_{s,m} + R_{v,m}$. By the above equations, we assume the population in each region is fully mixed, so the international travelers obey the distribution in their source region.

To combine the metapopulation epidemiological model with the global mobility network, we update each state of each region as follows:

$$S_n = S_n + \text{InputFlow}_{S_n}(t) - \text{OutputFlow}_{S_n}(t) = S_n + \sum_{m,m\neq n} mobility_{m,n}(t) \times \frac{S_m}{N_m} - \sum_{m,m\neq n} mobility_{n,m}(t) \times \frac{S_n}{N_n}, \tag{15}$$

$$V_n = V_n + \text{InputFlow}_{V_n}(t) - \text{OutputFlow}_{V_n}(t) = V_n + \sum_{m,m\neq n} mobility_{m,n}(t) \times \frac{V_m}{N_m} - \sum_{m,m\neq n} mobility_{n,m}(t) \times \frac{V_n}{N_n}, \tag{16}$$

$$I_{s,n} = I_{s,n} + \text{InputFlow}_{I_{s,n}}(t) - \text{OutputFlow}_{I_{s,n}}(t) = I_{s,n} + \sum_{m,m\neq n} mobility_{m,n}(t) \times \frac{I_{s,m}}{N_m} - \sum_{m,m\neq n} mobility_{n,m}(t) \times \frac{I_{s,n}}{N_n}, \tag{17}$$

$$I_{v,n} = I_{v,n} + \text{InputFlow}_{I_{v,n}}(t) - \text{OutputFlow}_{I_{v,n}}(t) = I_{v,n} + \sum_{m,m\neq n} mobility_{m,n}(t) \times \frac{I_{v,m}}{N_m} - \sum_{m,m\neq n} mobility_{n,m}(t) \times \frac{I_{v,n}}{N_n}, \tag{18}$$

$$R_{s,n} = R_{s,n} + \text{InputFlow}_{R_{s,n}}(t) - \text{OutputFlow}_{R_{s,n}}(t) = R_{s,n} + \sum_{m,m\neq n} mobility_{m,n}(t) \times \frac{R_{s,m}}{N_m} - \sum_{m,m\neq n} mobility_{n,m}(t) \times \frac{R_{s,n}}{N_n}, \tag{19}$$

$$R_{v,n} = R_{v,n} + \text{InputFlow}_{R_{v,n}}(t) - \text{OutputFlow}_{R_{v,n}}(t) = R_{v,n} + \sum_{m,m\neq n} mobility_{m,n}(t) \times \frac{R_{v,m}}{N_m} - \sum_{m,m\neq n} mobility_{n,m}(t) \times \frac{R_{v,n}}{N_n}. \tag{20}$$

Note that the number of deceased people $D_{s,n}, D_{v,n}$ will not be updated by the global mobility network.

During the model calibration, we use Bayesian Optimization (BO) with 40 random initializations to capture the underlying uncertainties that lie in the estimation of epidemiological parameters of the continuous mutation of SARS-CoV-2 virus. The metapopulation framework fits different sets of parameters for each region, accounting for the spatial heterogeneity of epidemiological parameters. Besides, we also divide the simulation period into three phases according to the mutation timeline, accounting for the temporal heterogeneity.

Leveraging the global flight data, we can reconstruct the global mobility pattern that contains other transportation methods, such as railway transport and maritime transport. According to two different industrial surveys from Statista and Aviation Benefits Beyond Borders, we assume our air travel data occupies 59% of global international travels. In that case, we reconstruct the global mobility pattern by multiplying $1/0.59$ for our model.

Text S3. Disentangling local transmission and imported transmission

We demonstrate the calculation process of the local transmission and imported transmission in Supplementary Fig 5. Assuming at the beginning of step t, we have a clear definition of the local transmission and imported transmission as the pre-existing infection cases locally and input cases through the global mobility network. Then for the new infections that happened at step t, we divide them by the proportion of local transmission and imported transmission, and add them back to the local transmission and imported transmission as an update. Then at step $t+1$, we have imported transmission from the last step, and new input cases from the global mobility network. We assume these two parts together are responsible for the imported transmission, while the others belong to local transmission. By tracing back the full transmission process, we can get the local transmission and imported transmission and determine the corresponding vaccine effectiveness towards them.

Text S4. Calibration of reported cases

Non-negligible errors due to the limited testing capacity and wrong statistic models have been identified[3,4], especially in regions with poor medical and health conditions. To minimize these errors, we leverage seroprevalence studies to calibrate the number of reported cases in Johns Hopkins University COVID-19 confirmed cases data.

We refer to the most comprehensive systematic review that screened 968 studies with more than 9.3 million participants in 74 countries[5], which is the largest synthesis of SARS-CoV-2 seroprevalence data. One of the major contributions of this review is the accurate assessment of the reliability of seroprevalence studies: leveraging Bayesian measurement error models, the authors corrected the seroprevalence estimates and labeled the reliability of each study. It successfully identified a large percentage of studies with insufficient reliability (496, 51% of all the studies). In our work, we preclude these unreliable studies

and adopt the corrected median seroprevalence provided by the authors, which had been cross-validated by Global Burden of Disease data maintained by the Institute for Health Metrics and Evaluation (IHME). We believe this estimation can minimize the potential bias and ensure the reliability of our following analyses. We calculate the calibration factors in each region by the reported infection rates and the median seroprevalence, which are further multiplied by the daily reported number. The detailed data are shown in Tab. SS5.

Text S5. Calculation of vaccine marginal utility

To investigate the effectiveness of vaccines in different regions, we calculate the marginal utility of vaccines by assigning additional 100 million vaccines in each region, and quantify the averted cases per vaccine 28 days later. We assign these additional vaccines to each region individually on 2021-10-17, and evaluate the difference between cumulative cases with and without these additional vaccines as an agent for the vaccine marginal utility. Besides, we also calculate the Spearman correlation between the vaccination rate on 2021-10-17 with the marginal utility in each region, finding that a strong negative correlation exists.

Supplementary Figures

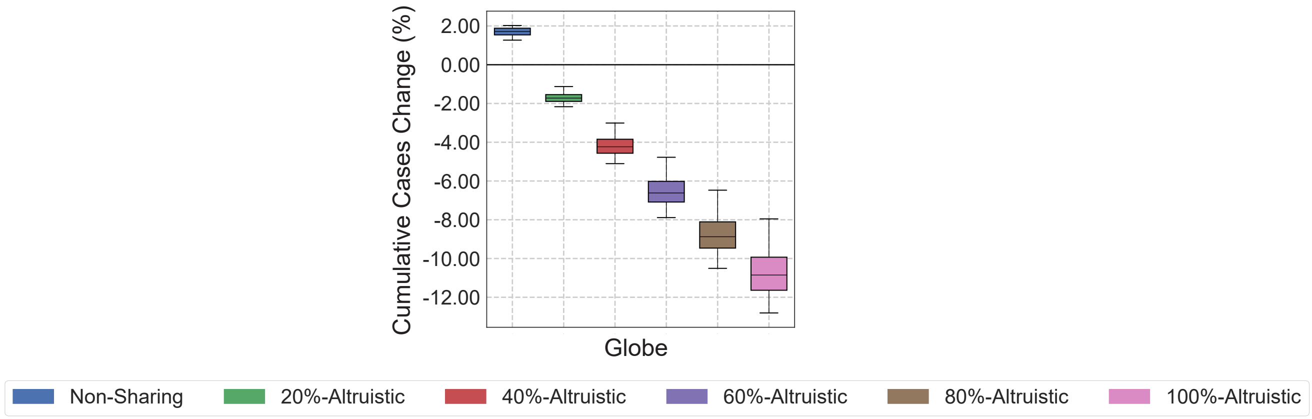

Supplementary Figure S1. Effectiveness of *altruistic* vaccine sharing strategies compared with *selfish* strategy. Under *altruistic* sharing strategy, vaccine-producing regions will start to share specific percentage of vaccines from the early beginning, without considering their own benefit. Although the global infections can be greatly suppressed, the *altruistic* strategies are not practical since they disobeys the interest of vaccine-producing regions.

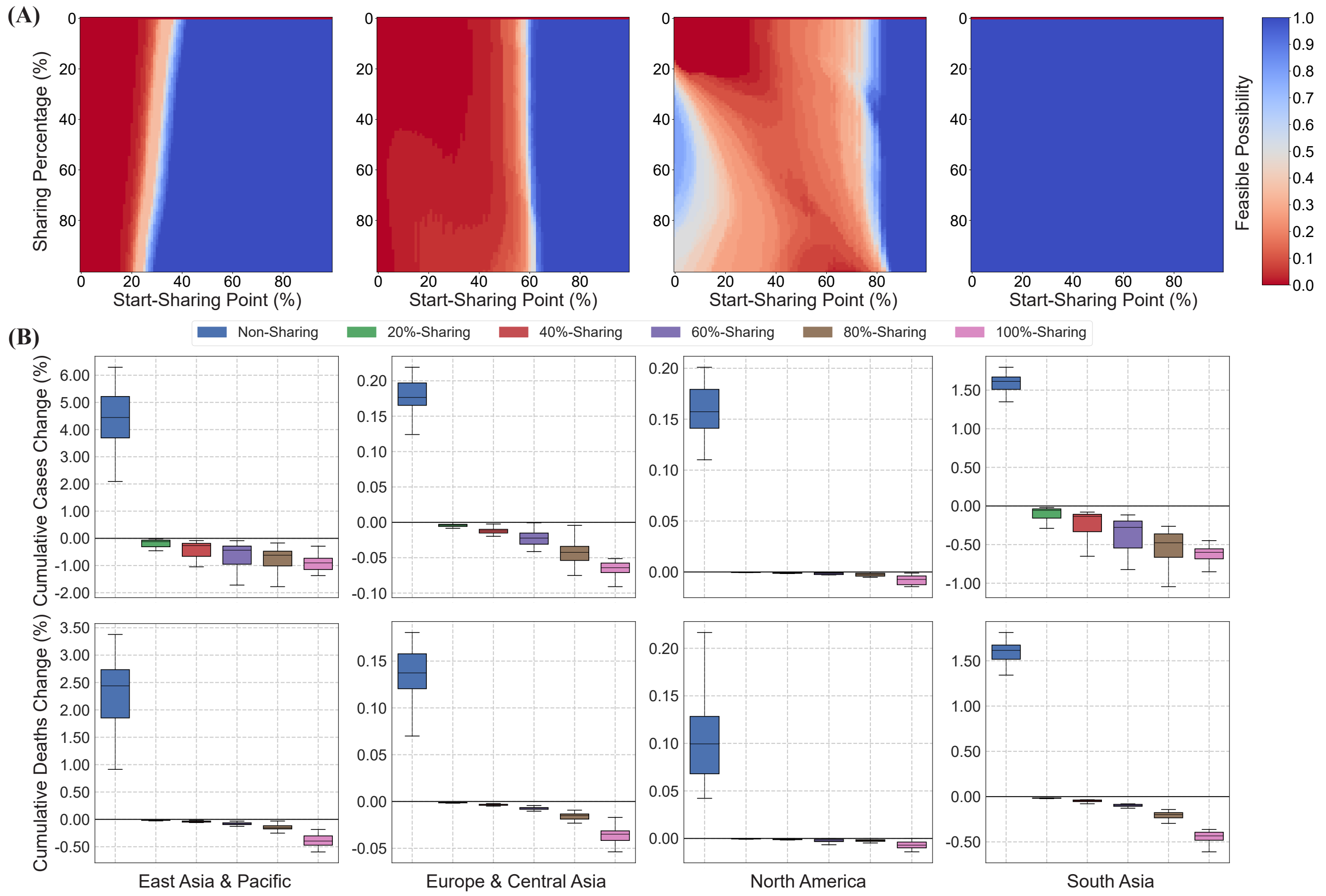

Supplementary Figure S2. Detailed possibilities of *enlightened self-interest*, statistics of cumulative cases change and cumulative deaths change of the four vaccine-producing regions. (A) The detailed possibility for each vaccine-producing region to have benefit with various vaccine sharing strategies over the repeated experiments where the color indicates the possibilities. **(B)** The detailed cumulative cases and deaths change comparing the *selfish* strategy in each vaccine-producing region.

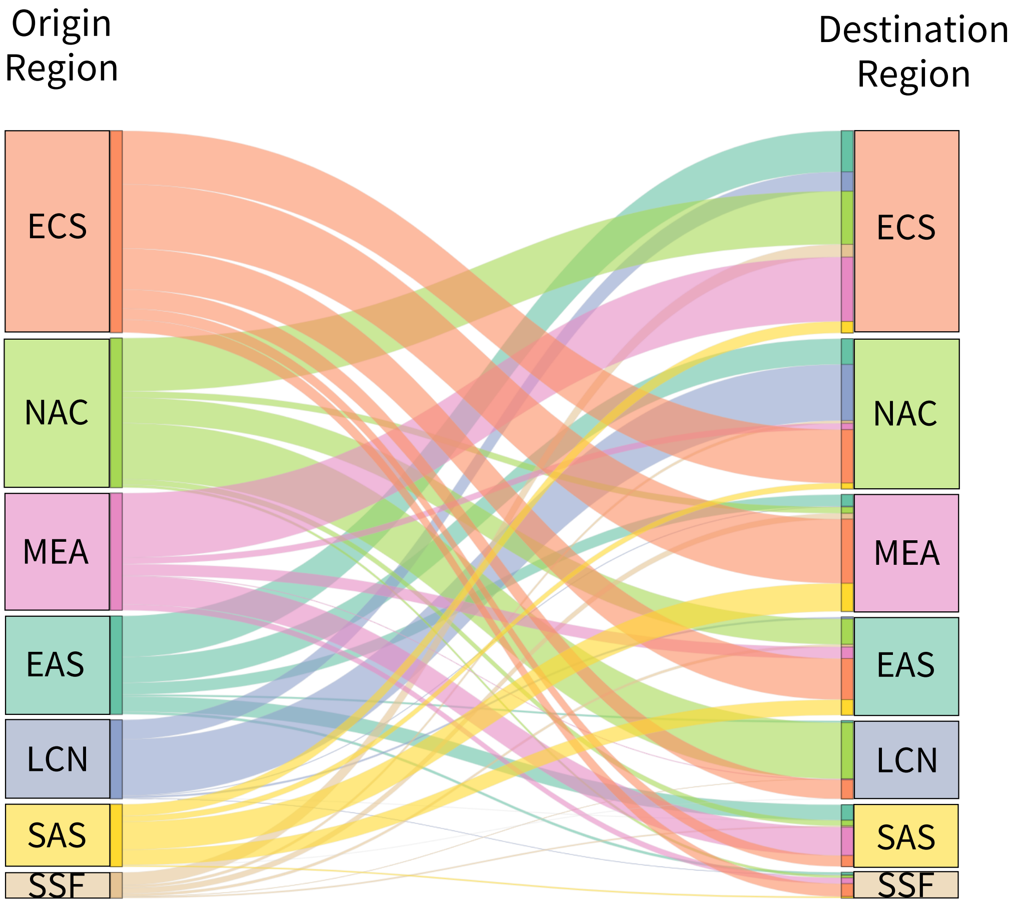

Supplementary Figure S3. Global population flow in region level. We demonstrate the cumulative population flow between regions during our simulation, which starts from 2020-03-01 to 2021-11-14. We assume the human mobility maintains the same level with 2019. From the figure we can observe highly heterogeneity in global mobility patterns, where the mobility is more concentrated in developed regions. While the population inflow and outflow are basically maintain an equilibrium.

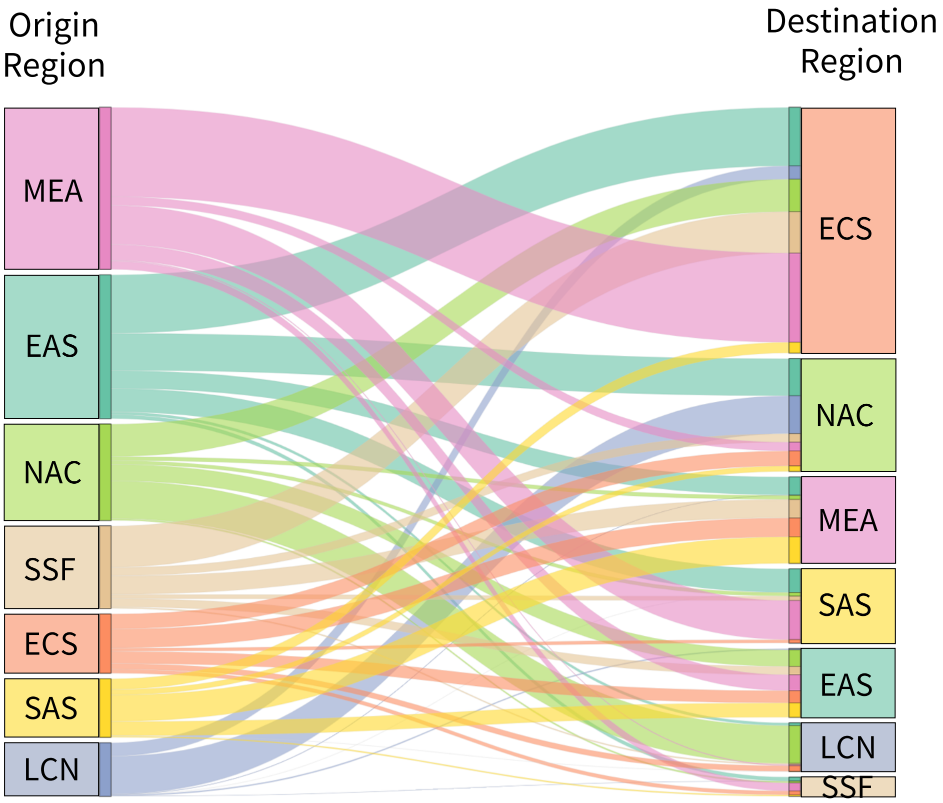

Supplementary Figure S4. Global population flow of infected people under no-vaccine scenario. If there are no vaccines, the flow of infected people demonstrates great heterogeneity: Middle East & North Africa will become the most important origin region, while Europe & Central Asia and North America will face great challenge from input infections.

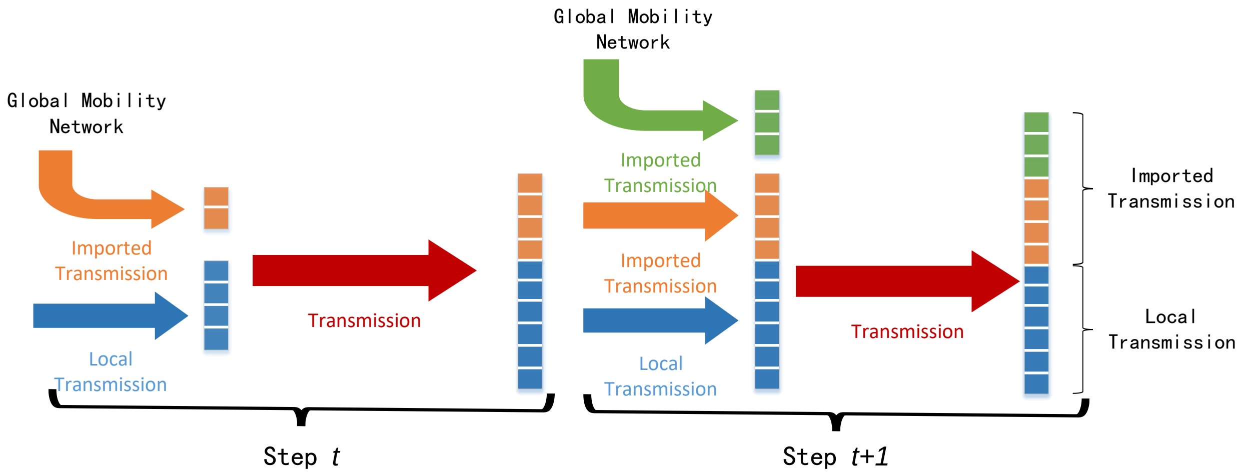

Supplementary Figure S5. Illustration of the definition about local transmission and imported transmission. The transmission can be divided into two types: the local transmission and the imported transmission. We trace back the new infections at each step according to the proportion of local infection and imported infection, where new infections caused by earlier local infections are defined as local transmission, and new infections caused by earlier and current input cases are defined as imported transmission.

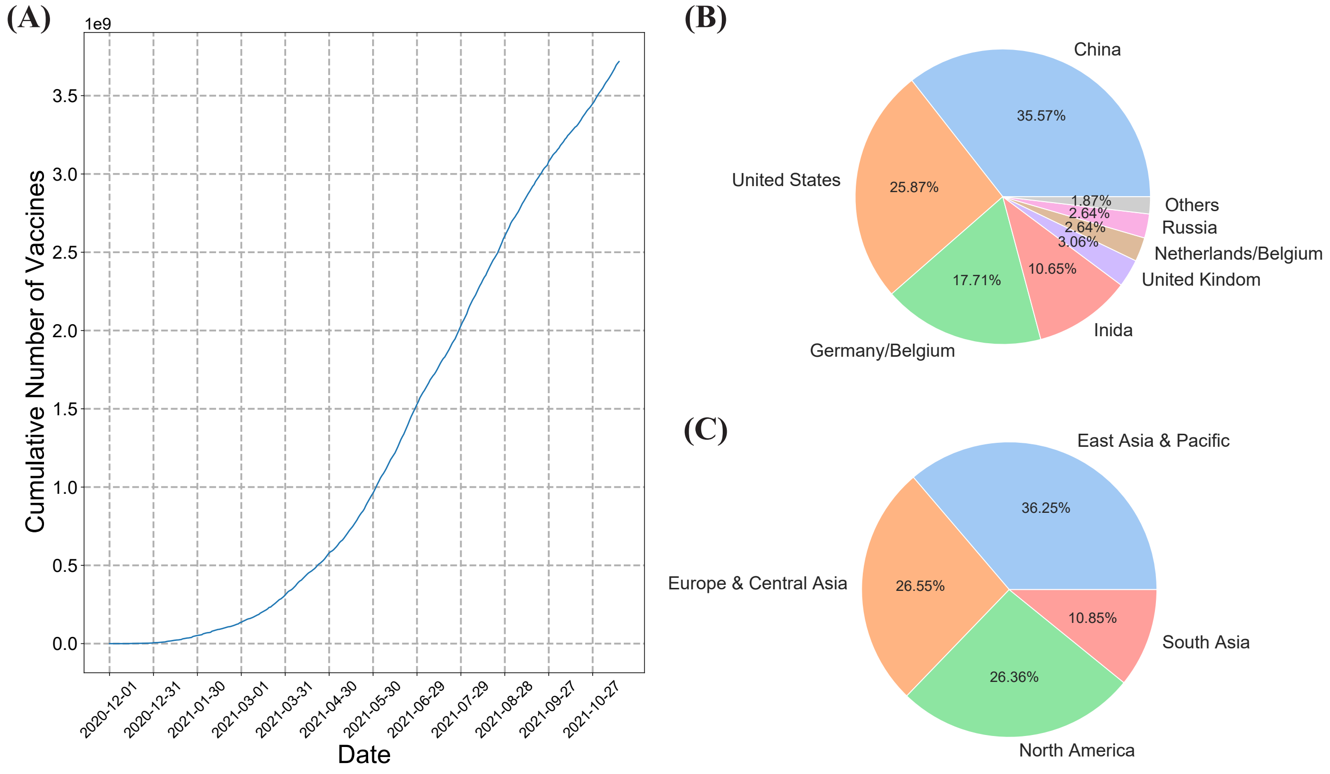

Supplementary Figure S6. Details in estimation of vaccine producing capacity. **(A)** The cumulative number of produced (administered) vaccines. **(B)** The shares of vaccine production in different countries until 2021-03-03. **(C)** The aggregated vaccine production shares into regions.

Supplementary Tables

Supplementary Table S1. Detailed information of the 183 studied countries (entities).

Country (Entity)	Region	Population[1]	Infections[2]	Vaccinations[3]
Afghanistan	South Asia	38928341	147154	5852810
Albania	Europe & Central Asia	2837743	133081	2000104
Algeria	Middle East & North Africa	43851043	171392	11316114
Andorra	Europe & Central Asia	77265	14678	104534
Angola	Sub-Saharan Africa	32866268	42777	7246966
Antigua and Barbuda	Latin America & Caribbean	97928	1303	111186
Argentina	Latin America & Caribbean	45376763	4929764	63017438
Armenia	Europe & Central Asia	2963234	230110	831856
Australia	East Asia & Pacific	25687041	34383	37695740
Austria	Europe & Central Asia	8917205	659056	12251544
Azerbaijan	Europe & Central Asia	10110116	343849	10092422
Bahamas	Latin America & Caribbean	393248	14840	263794
Bahrain	Middle East & North Africa	1701583	269186	2827532
Bangladesh	South Asia	164689383	1249484	84319490
Barbados	Latin America & Caribbean	287371	4390	283918
Belarus	Europe & Central Asia	9398861	446040	5409634
Belgium	Europe & Central Asia	11555997	1124715	16960398
Belize	Latin America & Caribbean	397621	14163	391846
Benin	Sub-Saharan Africa	12123198	8394	347270
Bhutan	South Asia	771612	2515	1148146
Bolivia	Latin America & Caribbean	11673029	473506	8041446
Bosnia and Herzegovina	Europe & Central Asia	3280815	205655	1553874
Botswana	Sub-Saharan Africa	2351625	106690	1128094
Brazil	Latin America & Caribbean	212559409	19917855	296865258
Brunei Darussalam	East Asia & Pacific	437483	337	680756
Bulgaria	Europe & Central Asia	6927288	425054	3070018
Burkina Faso	Sub-Saharan Africa	20903278	13573	661796
Burundi	Sub-Saharan Africa	11890781	7080	1084
Cabo Verde	Sub-Saharan Africa	555988	33791	526890
Cambodia	East Asia & Pacific	16718971	77243	28231176
Cameroon	Sub-Saharan Africa	26545864	82064	510324
Canada	North America	38005238	1438457	59539750
Central African Republic	Sub-Saharan Africa	4829764	7151	422372
Chad	Sub-Saharan Africa	16425859	4973	234030
Chile	Latin America & Caribbean	19116209	1615771	39036594
China	East Asia & Pacific	1402112000	105050	2389568000
Colombia	Latin America & Caribbean	50882884	4785320	50914404
Comoros	Sub-Saharan Africa	869595	4028	452612

Country (Entity)	Region	Population	Infections	Vaccinations
Congo	Sub-Saharan Africa	5518092	13186	549254
Congo (Democratic Republic)	Sub-Saharan Africa	89561404	49917	153616
Costa Rica	Latin America & Caribbean	5094114	406814	6736978
Cote d'Ivoire	Sub-Saharan Africa	26378275	50135	3541784
Croatia	Europe & Central Asia	4047200	363615	3845712
Cuba	Latin America & Caribbean	11326616	384596	27039584
Cyprus	Europe & Central Asia	1207361	101419	1237026
Czech Republic	Europe & Central Asia	10698896	1673576	12781462
Denmark	Europe & Central Asia	5831404	317790	8888556
Djibouti	Middle East & North Africa	988002	11651	92096
Dominica	Latin America & Caribbean	71991	210	53954
Dominican Republic	Latin America & Caribbean	10847904	341905	13565998
Ecuador	Latin America & Caribbean	17643060	487372	22692858
Egypt	Middle East & North Africa	102334403	284262	30563570
El Salvador	Latin America & Caribbean	6486201	86620	8888704
Equatorial Guinea	Sub-Saharan Africa	1402985	8880	438464
Estonia	Europe & Central Asia	1331057	133557	1459580
Eswatini	Sub-Saharan Africa	1160164	25979	286670
Ethiopia	Sub-Saharan Africa	114963583	280365	5066268
Fiji	East Asia & Pacific	896444	29781	1196888
Finland	Europe & Central Asia	5530719	106802	8223746
France	Europe & Central Asia	67391582	6190487	100781942
Gabon	Sub-Saharan Africa	2225728	25384	248310
Gambia	Sub-Saharan Africa	2416664	7709	267262
Georgia	Europe & Central Asia	3714000	419534	2046362
Germany	Europe & Central Asia	83240525	3776724	114930114
Ghana	Sub-Saharan Africa	31072945	103019	3188114
Greece	Europe & Central Asia	10715549	493304	13212742
Greenland	Europe & Central Asia	56367	119	77382
Grenada	Latin America & Caribbean	112519	164	70860
Guatemala	Latin America & Caribbean	16858333	368484	9152370
Guinea	Sub-Saharan Africa	13132792	25688	2364290
Guinea-Bissau	Sub-Saharan Africa	1967998	4479	307424
Guyana	Latin America & Caribbean	786559	22523	648974
Haiti	Latin America & Caribbean	11402533	20077	138330
Honduras	Latin America & Caribbean	9904608	297111	7584432
Hungary	Europe & Central Asia	9749763	809491	12958848
Iceland	Europe & Central Asia	366425	7959	581808
India	South Asia	1380004385	31655824	1122889436
Indonesia	East Asia & Pacific	273523621	3409658	214445104
Iran	Middle East & North Africa	83992953	3871008	98181400
Iraq	Middle East & North Africa	40222503	1626599	9632834
Ireland	Europe & Central Asia	4994724	300976	7358188
Israel	Middle East & North Africa	9216900	874018	16034098
Italy	Europe & Central Asia	59554023	4350028	92137032
Jamaica	Latin America & Caribbean	2961161	52895	1038132
Japan	East Asia & Pacific	125836021	927058	194370766
Jordan	Middle East & North Africa	10203140	770712	7662370
Kazakhstan	Europe & Central Asia	18754440	633469	16344892
Kenya	Sub-Saharan Africa	53771300	203213	5997816
Kiribati	East Asia & Pacific	119446	2	65278

Country (Entity)	Region	Population	Infections	Vaccinations
Kosovo	Europe & Central Asia	1775378	108365	1594448
Kuwait	Middle East & North Africa	4270563	397831	2668082
Kyrgyz Republic	Europe & Central Asia	6591600	162892	1863966
Laos	East Asia & Pacific	7275556	6299	4402770
Latvia	Europe & Central Asia	1901548	138863	2093452
Lebanon	Middle East & North Africa	6825442	561380	3443254
Lesotho	Sub-Saharan Africa	2142252	12908	383340
Liberia	Sub-Saharan Africa	5057677	5404	438562
Libya	Middle East & North Africa	6871287	249114	2096600
Liechtenstein	Europe & Central Asia	38137	3085	49388
Lithuania	Europe & Central Asia	2794700	282818	3497154
Luxembourg	Europe & Central Asia	632275	73870	856398
Madagascar	Sub-Saharan Africa	27691019	42663	566264
Malawi	Sub-Saharan Africa	19129955	52347	1304110
Malaysia	East Asia & Pacific	32365998	1113272	50989538
Maldives	South Asia	540542	77432	755588
Mali	Sub-Saharan Africa	20250834	14584	596434
Malta	Middle East & North Africa	525285	34295	906452
Mauritania	Sub-Saharan Africa	4649660	25691	1694010
Mauritius	Sub-Saharan Africa	1265740	3913	1764344
Mexico	Latin America & Caribbean	128932753	2848252	129806146
Moldova	Europe & Central Asia	2617820	259478	1567828
Monaco	Europe & Central Asia	39244	2889	49980
Mongolia	East Asia & Pacific	3278292	164155	4402206
Montenegro	Europe & Central Asia	621718	101927	514576
Morocco	Middle East & North Africa	36910558	623528	48083924
Mozambique	Sub-Saharan Africa	31255435	122028	7459438
Namibia	Sub-Saharan Africa	2540916	118922	614160
Nepal	South Asia	29136808	695389	16173246
Netherlands	Europe & Central Asia	17441139	1895343	24260870
New Caledonia	East Asia & Pacific	271960	134	340002
New Zealand	East Asia & Pacific	5084300	2874	7211962
Nicaragua	Latin America & Caribbean	6624554	9470	1674896
Niger	Sub-Saharan Africa	24206636	5623	896106
Nigeria	Sub-Saharan Africa	206139587	173908	8929326
North Macedonia	Europe & Central Asia	2083380	156380	1652784
Norway	Europe & Central Asia	5379475	137627	7991090
Oman	Middle East & North Africa	5106622	295857	5822656
Pakistan	South Asia	220892331	1034837	119035990
Panama	Latin America & Caribbean	4314768	435655	5514728
Papua New Guinea	East Asia & Pacific	8947027	17717	290512
Paraguay	Latin America & Caribbean	7132530	452388	5746120
Peru	Latin America & Caribbean	32971846	2111393	37772742
Philippines	East Asia & Pacific	109581085	1588965	69713994
Poland	Europe & Central Asia	37950802	2882939	39905294
Portugal	Europe & Central Asia	10305564	968631	16272250
Qatar	Middle East & North Africa	2881060	226239	4878412
Romania	Europe & Central Asia	19286123	1083189	13704556
Russia	Europe & Central Asia	144104080	6185249	114006988
Rwanda	Sub-Saharan Africa	12952209	70698	6765616
Samoa	East Asia & Pacific	198410	3	217716
San Marino	Europe & Central Asia	33938	5143	47502
Sao Tome and Principe	Sub-Saharan Africa	219161	2454	109686
Saudi Arabia	Middle East & North Africa	34813867	525730	46743788

Country (Entity)	Region	Population	Infections	Vaccinations
Senegal	Sub-Saharan Africa	16743930	62290	1961144
Serbia	Europe & Central Asia	6908224	721918	7365848
Seychelles	Sub-Saharan Africa	98462	18328	170530
Sierra Leone	Sub-Saharan Africa	7976985	6283	744826
Singapore	East Asia & Pacific	5685807	64981	10094498
Slovakia	Europe & Central Asia	5458827	392647	4858404
Slovenia	Europe & Central Asia	2100126	259215	2339018
Solomon Islands	East Asia & Pacific	686878	20	171562
Somalia	Sub-Saharan Africa	15893219	15403	691648
South Africa	Sub-Saharan Africa	59308690	2447454	23950944
South Korea	East Asia & Pacific	51780579	199787	81394840
South Sudan	Sub-Saharan Africa	11193729	11049	151244
Spain	Europe & Central Asia	47351567	4447044	73623626
Sri Lanka	South Asia	21919000	308812	29522460
Sudan	Sub-Saharan Africa	43849269	37138	1659666
Suriname	Latin America & Caribbean	586634	25351	463792
Sweden	Europe & Central Asia	10353442	1100040	14898408
Switzerland	Europe & Central Asia	8636896	717665	11279090
Syrian Arab Republic	Middle East & North Africa	17500657	25963	1125146
Tajikistan	Europe & Central Asia	9537642	15482	4852034
Tanzania	Sub-Saharan Africa	59734213	1017	1001610
Thailand	East Asia & Pacific	69799978	597287	83320620
Timor-Leste	East Asia & Pacific	1318442	10898	969618
Togo	Sub-Saharan Africa	8278737	15798	1412904
Trinidad and Tobago	Latin America & Caribbean	1399491	38811	1259500
Tunisia	Middle East & North Africa	11818618	589565	9858068
Turkey	Europe & Central Asia	84339067	5727045	118367034
Uganda	Sub-Saharan Africa	45741000	93927	3898698
Ukraine	Europe & Central Asia	44134693	2333409	20950876
United Arab Emirates	Middle East & North Africa	9890400	680858	21477304
United Kingdom	Europe & Central Asia	67215293	5883421	109179784
United States	North America	329484123	35037721	440559612
Uruguay	Latin America & Caribbean	3473727	381517	6694558
Uzbekistan	Europe & Central Asia	34232050	129327	29735466
Vanuatu	East Asia & Pacific	307150	4	114912
Venezuela	Latin America & Caribbean	28435943	305766	23156914
Vietnam	East Asia & Pacific	97338583	150060	98930570
Yemen	Middle East & North Africa	29825968	7061	547018
Zambia	Sub-Saharan Africa	18383956	195816	871880
Zimbabwe	Sub-Saharan Africa	14862927	108860	6165714

[1] Population data in 2020. For countries missing population data in 2020, the latest available data is used instead.
[2] Cumulative infections till 2021-07-31 according to Johns Hopkins University COVID-19 confirmed cases data.
[3] Cumulative vaccinations till 2021-11-16 according to Our World in Data COVID-19 vaccine doses administered data.

Supplementary Table S2. Detailed information of the 7 divided regions.

Region	Population[4]	Number of countries (entities)	Infections[5]	Infection Rate	Vaccines [6]	Vaccines per Capita
East Asia & Pacific (EAS)	2239251072	23	8499968	0.380%	3278819028	1.46
Europe & Central Asia (ECS)	917117307	53	59436989	6.48%	1076517888	1.17
Latin America & Caribbean (LCN)	648152307	30	40834286	6.30%	768626454	1.19
Middle East & North Africa (MEA)	459750854	20	12096249	2.63%	323960718	0.705
North America (NAC)	367489361	2	36476178	9.93%	500099362	1.36
South Asia (SAS)	1856882402	8	35171447	1.89%	1379697166	0.743
Sub-Saharan Africa (SSF)	1132500348	47	4772981	0.421%	108142914	0.0955
Total	7621143651	183	197288098	2.59%	7435863530	0.976

[1] Population data is obtained from World Bank Population, total. We calculate the population according to the 2020 data. For countries missing population data in 2020, the latest available data is used instead.
[2] Cumulative infections till 2021-07-31 according to Johns Hopkins University COVID-19 confirmed cases data.
[3] Cumulative vaccinations till 2021-11-16 according to Our World in Data COVID-19 vaccine doses administered data.

Supplementary Table S3. Income level statistics of each region.

Region	Income Group	#Country	Population	Country Percentage	Population Percentage
East Asia & Pacific (EAS)	High income	14	223450503	37.84%	9.56%
	Middle income	22	2088041938	59.46%	89.34%
	Low income	1	25778815	2.70%	1.10%
Europe & Central Asia (ECS)	High income	37	503240482	63.79%	54.50%
	Middle income	21	420211696	36.21%	45.5%
Latin America & Caribbean (LCN)	High income	16	28597416	39.02%	4.58%
	Middle income	25	595242966	60.98%	95.42%
Middle East & North Africa (MEA)	High income	8	68406280	38.10%	14.73%
	Middle income	11	348821218	52.38%	75.09%
	Low income	2	47326625	9.52%	10.19%
North America (NAC)	High income	3	367553264	100%	100%
South Asia (SAS)	Middle income	7	1817954061	87.50%	97.90%
	Low income	1	38928341	12.50%	2.10%
Sub-Saharan Africa (SSF)	High income	1	98462	2.08%	0.01%
	Middle income	24	582833059	50.00%	51.30%
	Low income	23	553115254	47.92%	48.69%

[1] Income group data is obtained from World Bank GDP (current US$).
[2] Population data is obtained from World Bank Population, total. We calculate the population according to the 2020 data.

Supplementary Table S4. Epidemiological parameters of our global mobility-aware SIR model.

Name	Description	Value
Infection rate (β)	Probability of getting infected when a susceptible person collocates with an exposed or infected person. We assume the transmission probability is equal for exposed and infected people.	Learnable parameter in three stages
Recovery rate (γ)	The rate with which infected individuals recover or die.	Learnable parameter in three stages
Global average death rate (ψ)	The death rate inferred from real-world infection fatality rate (IFR) calculated by John Hopkins statistic on 2022-02-28	1.4%
Vaccine effectiveness towards infection (κ)	The percentage of vaccinated people that will not get infected by COVID-19	85%[15–17]
Vaccine effectiveness towards death (κ)	The percentage of infected, vaccinated people that will not die because of COVID-19	90%[15–17]

Supplementary Table S5. Detailed data in calibration of reported cases.

Region	Reported infections	Reported infection rate (%)	Median seroprevalence (%)	Calibration factor
East Asia & Pacific	1823286	0.082	1.0	12.215
Europe & Central Asia	25265184	2.885	5.3	1.837
Latin America & Caribbean	15447574	2.546	6.8	2.671
Middle East & North Africa	4227264	0.980	12.9	13.163
North America	20740865	5.644	3.8	(1.000)[5]
South Asia	11653046	0.628	17.6	28.045
Sub-Saharan Africa	1783721	0.193	14.6	75.592

[5] North America shows higher reported infection rate than median seroprevalence and thus we leave the number of reported infection uncalibrated, i.e., the calibration factor is 1.000.

Supplementary Table S6. Calibrated parameters in our model.

Region	β			γ		
	Phase 1	Phase 2	Phase 3	Phase 1	Phase 2	Phase 3
East Asia & Pacific	1.351	0.314	0.203	0.813	0.276	0.068
Europe & Central Asia	1.974	0.695	0.671	0	0.536	0.624
Latin America & Caribbean	1.351	0.314	0.203	0.813	0.276	0.0684
Middle East & North Africa	1.732	0.536	0.478	0.746	0.519	0.357
North America	2.010	0.255	0.226	0	0.193	0.152
South Asia	1.697	0.870	0.946	0.666	0.783	0.499
Sub-Saharan Africa	1.049	0.099	0.069	0.177	0.058	0.028

References

1. Johns Hopkins University Center for Systems Science and Engineering. 2019 novel coronavirus COVID-19 (2019-nCoV) data repository by johns hopkins CSSE (2020). https://github.com/CSSEGISandData/COVID-19 (2020).
2. World Bank Open Data. https://data.worldbank.org.
3. Van Noorden, R. COVID death tolls: scientists acknowledge errors in WHO estimates. *Nature* **606**, 242–244 (2022).
4. Perkins, T. A. *et al.* Estimating unobserved SARS-CoV-2 infections in the United States. *Proc. Natl. Acad. Sci.* **117**, 22597–22602 (2020).
5. Bobrovitz, N. *et al.* Global seroprevalence of sars-cov-2 antibodies: a systematic review and meta-analysis. *PloS one* **16**, e0252617 (2021).
6. Our World in Data. https://ourworldindata.org.
7. Gozzi, N. *et al.* Estimating the impact of COVID-19 vaccine inequities: a modeling study. *Nature Communications* **14**, 3272 (2023).
8. Karaivanov, A., Kim, D., Lu, S. E. & Shigeoka, H. COVID-19 vaccination mandates and vaccine uptake. *Nature Human Behaviour* **6**, 1615–1624 (2022).
9. Statista. https://www.statista.com.
10. Wu, J. T., Leung, K. & Leung, G. M. Nowcasting and forecasting the potential domestic and international spread of the 2019-nCoV outbreak originating in Wuhan, China: a modelling study. *The Lancet* **395**, 689–697 (2020).
11. Zhong, L., Diagne, M., Wang, W. & Gao, J. Country distancing increase reveals the effectiveness of travel restrictions in stopping COVID-19 transmission. *Communications Physics* **4**, 121 (2021).
12. Colizza, V., Barrat, A., Barthelemy, M., Valleron, A.-J. & Vespignani, A. Modeling the worldwide spread of pandemic influenza: baseline case and containment interventions. *PLOS Medicine* **4**, e13 (2007).
13. Menkir, T. F. *et al.* Estimating internationally imported cases during the early COVID-19 pandemic. *Nature Communications* **12**, 311 (2021).
14. Lemey, P. *et al.* Untangling introductions and persistence in COVID-19 resurgence in Europe. *Nature* **595**, 713–717 (2021).
15. Nasreen, S. *et al.* Effectiveness of COVID-19 vaccines against symptomatic SARS-CoV-2 infection and severe outcomes with variants of concern in ontario. *Nat. Microbiol.* 1–7 (2022).
16. Dagan, N. *et al.* BNT162b2 mRNA Covid-19 vaccine in a nationwide mass vaccination setting. *New Engl. J. Medicine* (2021).
17. Chodick, G. *et al.* The effectiveness of the TWO-DOSE BNT162b2 vaccine: analysis of real-world data. *Clin. Infect. Dis.* **74**, 472–478 (2022).
